# The Role of Metabolism in Migraine Pathophysiology and Susceptibility

**DOI:** 10.3390/life11050415

**Published:** 2021-05-01

**Authors:** Olivia Grech, Susan P. Mollan, Benjamin R. Wakerley, Daniel Fulton, Gareth G. Lavery, Alexandra J. Sinclair

**Affiliations:** 1 Metabolic Neurology, Institute of Metabolism and Systems Research, College of Medical and Dental Sciences, University of Birmingham, Birmingham B15 2TT, UK; oxg958@student.bham.ac.uk (O.G.); b.wakerley@bham.ac.uk (B.R.W.); g.g.lavery@bham.ac.uk (G.G.L.); 2 Centre for Endocrinology, Diabetes and Metabolism, Birmingham Health Partners, Birmingham B15 2TH, UK; 3 Birmingham Neuro-Ophthalmology Unit, University Hospitals Birmingham NHS Foundation Trust, Birmingham B15 2TH, UK; soozmollan@doctors.org.uk; 4 Department of Neurology, Queen Elizabeth Hospital, University Hospitals Birmingham NHS Trust, Birmingham B15 2TH, UK; 5 Institute of Inflammation and Ageing, University of Birmingham, Birmingham B15 2TT, UK; d.fulton@bham.ac.uk

**Keywords:** migraine, migraine with aura, metabolism, cortical spreading depression

## Abstract

Migraine is a highly prevalent and disabling primary headache disorder, however its pathophysiology remains unclear, hindering successful treatment. A number of key secondary headache disorders have headaches that mimic migraine. Evidence has suggested a role of mitochondrial dysfunction and an imbalance between energetic supply and demand that may contribute towards migraine susceptibility. Targeting these deficits with nutraceutical supplementation may provide an additional adjunctive therapy. Neuroimaging techniques have demonstrated a metabolic phenotype in migraine similar to mitochondrial cytopathies, featuring reduced free energy availability and increased metabolic rate. This is reciprocated in vivo when modelling a fundamental mechanism of migraine aura, cortical spreading depression. Trials assessing nutraceuticals successful in the treatment of mitochondrial cytopathies including magnesium, coenzyme q10 and riboflavin have also been conducted in migraine. Although promising results have emerged from nutraceutical trials in patients with levels of minerals or vitamins below a critical threshold, they are confounded by lacking control groups or cohorts that are not large enough to be representative. Energetic imbalance in migraine may be relevant in driving the tissue towards maximum metabolic capacity, leaving the brain lacking in free energy. Personalised medicine considering an individual’s deficiencies may provide an approach to ameliorate migraine.

## 1. Introduction

### 1.1. Background

Headache disorders are ranked the second most prevalent disease worldwide [1], with migraine in particular affecting 1 billion people [2]. Migraine is also the leading cause of disability amongst neurological disorders [3], and significantly reduces quality of life [4]. It severely disrupts sufferers personal [5] and work-related functionality [6] and is estimated to cost €93 billion in Europe, due to both health costs and lost productivity [7]. However, despite its significant prevalence and disability, the importance of migraine as a public health issue has only recently been recognised. Moreover, migraine remains underdiagnosed and often untreated [8], with the most commonly recommended treatment triptans only effective in ~60% of patients [9]. The relationship between episodic (<15 days with migraine per month) and chronic (≥15 days per month) migraine [10] is complex and there remains a crucial gap regarding the pathophysiology of headache, preventing the development of effective targeted drugs.

### 1.2. Headache Mechanisms

Research has since evolved from early concepts that migraine is solely vasculature driven [11], instead considering complex nociceptive structures and tissue excitability in its pathogenesis. Activation of the trigeminovascular system, consisting of sensory trigeminal nerve fibers which innervate cerebral blood vessels and dura mater, has long been hypothesised to underlie headache pain [12]. Prolonged central sensitisation of the system leads to hyperexcitability of trigeminal neurons and decrease in nociceptive threshold, which is hypothesised to drive the transition from episodic to chronic migraine [13]. Of importance is the neuropeptide calcitonin gene-related peptide (CGRP), which is both released and has receptors throughout the trigeminovascular system [14]. Clinical studies have demonstrated the causative role of CGRP in migraine by measuring increased levels in the circulation during attacks [15], and recording headache onset following CGRP infusion [16].

Migraine with aura is a subtype which features visual, sensory or central nervous system symptoms which often precede headache attack [10]. These symptoms are accompanied by a decrease in regional blood flow in the cortex and cortical spreading depression (CSD) [10,17]. CSD is a wave of depolarisation which propagates slowly (2 to 5 mm/min) across the cerebral cortex [18]. This leads to neurovascular changes, influx of Na^+^ and efflux of K^+^ and release of neuropeptides, further propagating the spread of depression [18,19]. These vast changes in tissue excitability are followed by a period of suppressed stimulated and spontaneous synaptic activity, termed neuronal silencing [20]. In addition to aura, clinical evidence has also associated CSD with traumatic brain injury, stroke and subarachnoid haemorrhage [17,21,22]. Animal studies have demonstrated the ability of CSD to activate the trigeminovascular system, particularly by its ability to release CGRP [23], implicating CSD in headache generation [24]. Emerging migraine therapeutics aim to target these mechanisms and include monoclonal antibodies and receptor antagonists. These aim to block either CGRP itself or its receptor and have provided effective pain relief in migraine [25,26,27,28,29].

There are a number of secondary headache conditions whose headache phenotype mimics migraine, such as post traumatic headache [30] and persistent post idiopathic intracranial hypertension (IIH) headache [31]. Headache in IIH is driven by raised intracranial pressure and reduction of intracranial pressure has been reported in some studies to reduce headache [10]. Despite resolution of papilledema and normalization of raised intracranial pressure, many suffer persistent post-IIH headache that imitates migraine [32]. A recent open label study of persistent post-IIH headaches evaluated the use of Erenumab, a monoclonal antibody that binds the CGRP ligand. CGRP therapy reduced frequency of monthly moderate/severe headache days by 71% and all headache days by 45% from baseline to 12 months [32]. This study brought new insights to the association of CGRP in IIH headache. Seven of the patients had a recurrence of their raised intracranial pressure (evidenced by papilloedema), yet no return of their headache, suggesting CGRP may have a role in headache attributed to IIH [33]. Similarly, CGRP was found to induce headache exacerbation with migraine-like features in patients with persistent post-traumatic headache attributed to mild traumatic brain injury in a recent randomized control trial [34]. The understanding of the biological underpinnings for conditions that mimic migraine are intriguing, where some have suggested shared biological foundations [35].

### 1.3. Energy Metabolism in Headache

A mismatch between brain energy supply and demand has also been hypothesised to contribute toward headache pathology, with many studies suggesting fundamental dysfunction of mitochondria [36]. Despite multiple clinical trials assessing the use of nutraceuticals to target this and support metabolic processes, these often lack suitable control groups, and the results remain contradictory [37,38]. Identifying specific dysfunctional metabolic pathways and targets has hampered the progress of therapeutic development. Although a multitude of metabolic and respiratory pathways are associated with headache disorders, this review will focus specifically on aspects which are identified as dysfunctional in migraine. This review will discuss the clinical and pre-clinical evidence evaluating metabolic perturbations with potential therapeutic value for migraine, and secondary headaches which mimic migraine, such as IIH.

## 2. Patient Studies

### 2.1. ATP-PCR System

**Figure 1:**
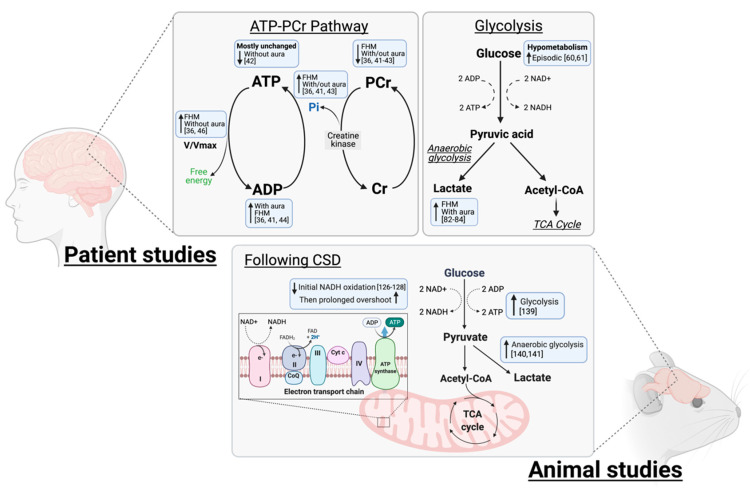
Perturbations in metabolic pathways as demonstrated in patient and animal studies. Neuroimaging studies in patients have exhibited disturbances in the ATP-PCR pathway which indicate a low free energy availability coupled with an increased metabolic rate. Glucose hypometabolism has been noted in migraine patients in addition to increased lactate, a product of anaerobic glycolysis. In animal studies however glycolysis is increased due to the rapid increase in ATP demand following headache mechanisms. This rapid incline also results in rapid NADH oxidation, which may lead to tissue hypoxia and anaerobic respiration.

ATP is the universal energy storage molecule and is mostly generated in the brain by oxidative phosphorylation of ADP in mitochondria. This is coupled to creatine kinase reactions which donate phosphate (Pi) from phosphocreatine (PCr) to ADP to re-synthesise ATP from ADP in the ATP-PCR system (Figure 1). This system allows rapid mobilization of limited high energy phosphates to regenerate ATP during metabolic stress. Phosphorus-31 nuclear magnetic resonance spectroscopy (^31^P-MRS) is a non-invasive tool capable of quantifying phosphorus-containing compounds including ATP and PCr in vivo in the brain [39]. It is also possible to calculate additional parameters such as ADP concentration, phosphorylation potential and V/Vmax (actual velocity of oxidative metabolism/the maximum oxidative capacity) [40].

^31^P-MRS studies have overwhelmingly highlighted aspects of mitochondrial dysfunction in migraine [40]. PCr content, indicative of free cellular energy, is significantly decreased at rest in the brain of familial hemiplegic migraine (FHM) and both migraine with and without aura patients [36,41,42]. Decreased PCr was also exhibited during migraine attacks in patients with aura [43] and in muscle following exercise in FHM patients [36], suggesting a systemic mitochondrial dysfunction. Increase in Pi is also demonstrated both between and during migraine attacks [36,41,43], resulting in a decreased PCr/Pi ratio [43,44,45]. Increased ADP [36,41,44] and percentage of V/Vmax during interictal phases is another consistent finding in migraine [36,46], indicating an increased metabolic rate and oxidative capacity, resulting in a lower energy reserve. In most studies, ATP concentration remained similar between patients and controls, except for Reyngoudt et al., who identified a significant decrease in ATP in migraine without aura patients [42]. To summarise, ^31^P-MRS findings in migraine patients indicate an imbalance between increased brain metabolism and decrease in free cellular energy availability, which has been hypothesised as a biochemical substrate for headache attack [42,47]. This pattern is typical of defective mitochondrial respiration with low PCr, high Pi and high ADP found in mitochondrial cytopathies [48], which hints that migraine may also share similar aspects of pathology.

### 2.2. Magnesium Availability

^31^P-MRS is also able to measure free cytosolic magnesium (Mg^2+^), an important coenzyme in the creatine kinase reaction. Mg^2+^ may have multiple targets in headache; low concentrations have been associated with spontaneous CSD [49], it is able to induce changes in vascular tone [50], and influence neurotransmitter release [51]. Reduced Mg^2+^ content has been exhibited both during headache attacks and interictal periods in migraine [52,53]. Decreased Mg^2+^ was also associated with reduced free energy released from ATP hydrolysis in several migraine subtypes [52]. Notably, reductions showed a trend in line with severity of clinical phenotype, with the lowest measurements in patients with migraine associated with stroke and highest in migraine without aura [52].

In addition to neuroimaging, direct measurements of serum Mg^2+^ have exhibited significant reductions during interictal periods in migraine patients compared to controls [54,55]. Total serum Mg^2+^ exhibited a negative linear relationship with migraine attack frequency [54], and a further reduction during attack [55]. Although this may suggest systemic Mg^2+^ deficiency, accurate testing is difficult to achieve due its compartmentalisation and absorption in the body, and there remains debate as to which form of Mg^2+^ to measure. In a study of chronic daily headache, ionized Mg^2+^ was significantly decreased in serum compared to controls, although there were no differences in total Mg^2+^ [56].

Studies attempting to assess the efficacy of Mg^2+^ for the treatment of headache have demonstrated conflicting results. Some have exhibited a prophylactic effect of oral Mg^2+^ (486–600 mg) at reducing migraine attack frequency and duration [57,58,59] whilst intravenous magnesium sulfate (1 g) demonstrated reduction in pain [37,60]. However, some trials proving its efficacy have been confounded by the lack of a control group, are not large enough to be conclusive [60,61], or have focused on a sub-cohorts of patients with lower serum Mg^2+^ [61]. Additionally a double-blind placebo-controlled study of oral Mg^2+^ observed no effect on migraine after 12 weeks treatment [59], and emergency department use of intravenous Mg^2+^ has demonstrated, in multiple studies, no significant pain relief [38,62]. Discrepancies in the effectiveness of Mg^2+^ treatment between trials could be due to variance in the serum levels of Mg^2+^ of participants, or route of administration [37,61]. Therefore, preliminary screening could identify patients who will better benefit from nutraceutical treatment, as those with ionised serum Mg^2+^ > 0.54 mmol/L did not respond to therapy. [61] Overall the current evidence does not convincingly demonstrate that Mg^2+^ in patients interacts with the pathophysiology of migraine.

### 2.3. Glycolysis and Glucose Metabolism

Metabolically challenging events such as fasting and exercise are established triggers of migraine attacks, further implicating the role of metabolism [63,64]. Migraineurs have demonstrated both impaired insulin sensitivity and higher fasting plasma insulin during interictal periods [65,66], with reduced insulin release during attacks [67]. Therefore, the metabolism of glucose has been of significant interest in migraine pathology.

18F-Fluorodeoxyglucose PET (18F-FDG PET) imaging allows the measurement of localised cerebral glucose metabolism with use of a radiotracer-labelled glucose analogue. Interictal periods in episodic migraineurs exhibited significant glucose hypometabolism in several regions involved in central pain processing, in comparison to controls [68]. A negative correlation was found between disease duration and lifetime headache frequency with glucose metabolism in the insula and anterior cingulate cortex. These results suggest repeated migraine attacks over time lead to progressive decline in glucose metabolism of central pain processes [68]. This is a similar finding in medication overuse headache (MOH) patients who exhibit hypometabolism in pain processing regions [69].

These perturbations, however, are not fixed and appear reversible with treatment. Hypometabolism in fronto-temporal areas in episodic migraineurs improved following three months of external trigeminal nerve stimulation, which also significantly decreased frequency of migraine attacks [70]. Brain regions recovered to almost normal glucose uptake following withdrawal from analgesics in MOH in all regions except the orbitofrontal cortex [69]. Although the underlying cause of hypometabolism in relation to migraine pathology remains unknown, these studies suggest that improving glucose utilisation may increase the threshold for sensitization in pain processing structures or prevent induction of migraine generating mechanisms.

### 2.4. Ketogenesis

In the absence of glucose and glycogen stores, ketone bodies are produced from fatty acids by astrocytes to serve as energetic substrate in a metabolic pathway known as ketogenesis. [71]. The ketogenic diet (low carbohydrates <50 mg/day [72]) is able to induce this pathway and has been effective for the treatment of severe epilepsy [73]. Given that tissue excitability is also altered in migraine, it has been hypothesised that this diet may be favourable at restoring brain metabolism and excitability in migraine pathophysiology [72].

Small (1–2 patients) case reports have noted migraine improvement in those on a ketogenic diet [74,75] and a proof-of-concept study demonstrated a significant reduction in attack frequency and number of days with headaches during the first month of ketogenesis [76]. A recent study observed alterations in cortical excitability as measured by visual and somatosensory-evoked potentials, in addition to reduced attack frequency and duration following one month of dieting [77]. However, the efficacy of this diet remains under scrutiny [78], and a pilot study of the modified Atkins diet (high-fat low-carbohydrates) for chronic daily headache in adolescents demonstrated no protective effect [79]. Moreover, although studies have demonstrated initial positive effects, clinical variables appear to worsen following the initial month of dieting, after which patients are out of the ketogenic phase [76]. There has been no strong evidence to outline the most conclusive diet for migraine prophylaxis and it is yet to be determined if it is the weight loss which is effective rather than the components of the diet.

### 2.5. Anaerobic Metabolism

Hypoxia contributes towards headache in disorders such as high-altitude headache and obstructive sleep apnoea [10]; however, it has also been hypothesised to trigger migraine attack [80,81]. Therefore, measuring anaerobic products of glycolysis including lactic acid (Figure 1) can provide insights into the role of hypoxia in migraine triggers and pathology.

Proton MRS (1H-MRS) is also a non-invasive imaging method which can provide measurements of neurotransmitters and metabolites including lactate [40]. This method has demonstrated elevated lactate in the brain during interictal periods in FHM [82] and migraine with aura [83,84]. It is thought that transient increases in lactate at rest and in the absence of hypoxia, may indicate a subtle mitochondrial dysfunction and aberrant glycolysis. These results, however, are controversial as lactate is endogenously present in low concentrations in healthy brain tissue, making subtle variations difficult to measure. Higher field strength (3T) 1H-MRS studies, measuring absolute rather than relative concentrations of metabolites, did not detect a significant increase in lactate in migraine without aura patients, in comparison to controls [85]. Furthermore, in a provocation study, normobaric hypoxia was able to induce migraine in aura patients and although lactate did increase, it was not significantly higher than in controls [80].

Direct measurements of lactate in blood or cerebrospinal fluid (CSF) have also demonstrated conflicting results in migraine. There have been key associations between lactate and CSD, with a marked increase in lactate/pyruvate ratio following 12 temporally grouped CSDs in a migraine-associated stroke patient [86]. However, there are discrepancies between lactate measurements from different sample types. For instance, significantly higher lactic acid concentrations have been exhibited in blood, serum and plasma for migraine [87,88], but only serum for tension-type headache [88,89]. Increased lactate measurements may be indicative of cerebral hypoxia and shifts to anaerobic respiratory processes. Therefore, it may be anticipated that pH in the brain tissue would decrease as a result of lactic-acidosis, however, this is not found in NMR studies [90].

### 2.6. Prophylactic Supplementation of Patients

In response to the evidence of metabolic dysfunction, there have been a plethora of trials assessing the efficacy of dietary supplements and nutraceuticals for headache prophylaxis. Coenzyme Q10 (CoQ10) is a key co-factor of the electron transport chain which has mostly been used in mitochondrial cytopathies to improve dysfunctional respiratory metabolism. Supplementation was able to reduce CSF lactate and pyruvate levels in mitochondrial encephalopathy, lactic acidosis, and stroke-like episodes (MELAS) [91] and ^31^P-MRS studies have demonstrated improvement in mitochondrial respiration. [92] Several emerging trials have also validated CoQ10′s efficacy in migraine, with oral supplementation (150–300 mg) capable of reducing migraine-attack frequency [93] with 50% reduction after 3 months [94,95] and reducing severity [94]. In addition to its role in mitochondrial respiration, CoQ10 also has an antioxidant and anti-inflammatory role, which would be beneficial in its treatment of headache. Supplementation in episodic migraine patients reduced inflammatory mediator TNF-α and CGRP, both thought to be involved in trigeminal sensitisation [96]. Although deficiency has been exhibited in juvenile cases of migraine [97], it is yet to be well documented in adults, but could illustrate a dysfunctional component of the mitochondrial respiratory chain.

Riboflavin (vitamin B2) is the precursor of flavin mononucleotide (FMN), a component of complex I, and flavin adenine dinucleotide (FAD) an electron donor in the mitochondrial respiratory chain. Riboflavin is able to reduce headache pain in MELAS, in which it is hypothesised to support complex I and II activity in the electron transport chain. [98] In migraine, treatment with oral riboflavin (400 mg) was able to reduce attack frequency [99] and severity [99,100], hinting towards shared pathology with MELAS.

There has been accumulating interest for the role of vitamin D in migraine, with several studies reporting low serum levels in migraine patients [101,102,103,104]. Moreover, the incidence of aura, phono/photophobia and resistance to medication was found to be significantly higher in migraine patients with a deficiency compared to those with normal levels [105]. Supplementation with vitamin D in migraine has demonstrated the ability to reduce headache frequency, headache diary result and migraine disability score [101,106,107]. Although the mechanism of vitamin D has remained elusive, a recent study revealed a significant reduction in serum CGRP concentration following 16 weeks of supplementation [107]. Future larger randomized control trials are needed to further investigate the role of vitamin D, as few studies have contradicted the findings of deficiency in migraine [108].

Palmitoylethanolamide (PEA) is an endogenous fatty acid amide abundant throughout the body including the central nervous system. It has analgesic and anti-inflammatory properties mediated by its binding to the peroxisome proliferator-activated receptor α (PPAR- α) [109,110,111]. PPAR- α also acts as a ligand binding transcription factor and stimulates fatty acid oxidation, [112] and PEA supplementation in animal models is able to modulate hepatic mitochondrial oxidative capacity [113] and glycolytic potential [114]. PEA has been effective at reducing pain in conditions of chronic pain and inflammation [115]; therefore it has become a nutraceutical of interest in migraine. In migraine with aura patients, PEA (1200 mg) and NSAIDs were able to significantly reduce pain intensity after 60 days in comparison to a group that received NSAIDs alone [116]. Similarly PEA was able to reduce headache frequency and intensity in a paediatric population with migraine without aura [117]. Use of nutraceuticals may be a useful additional headache therapy with a low side effect profile. Considering studies which have highlighted metabolite deficiencies, nutraceuticals may also pose as a personalised medicine dependant on an individual’s insufficiency.

## 3. Animal Data

Preclinical animal models have been invaluable in clarifying the pathophysiology of headache in addition to identifying therapeutic targets [118,119]. These models include mice expressing familial migraine mutations [120,121], models which sensitise the trigeminal system or directly induce CSD [122]. Studying metabolism in vivo is advantageous as respiratory processes can be easily manipulated and challenged, whilst more invasive biochemical methods can be utilised. Although direct measurements of pain cannot be achieved in rodents, appropriate behavioural studies can be used to provide a readout of this important clinical feature of headache [123].

### 3.1. Imaging and Labelling Studies

#### 3.1.1. NADH and Oxidative Metabolism

Techniques using animal models benefit from mitochondrial respiration measurements in real-time and in vivo. Two-photon fluorescence imaging utilises the autofluoroscent reduced form of NADH, which donates an electron in the mitochondrial respiratory chain to become the oxidised and non-fluorescent NAD+ (Figure 1). Measuring NADH fluorescence in vivo allows the assessment of changes in mitochondrial redox potential, in addition to the occurrence of metabolically limiting hypoxia [124,125].

Following the widespread depolarisation of neurons and glia involved in CSD, there is a surge in ATP demand in cortical regions as the affected cells attempt to restore ionic gradients via ATPase Na^+^/K^+^ pumps [18]. This has established CSD as a metabolically demanding event. Multiple studies utilising NADH fluorescence measurements have characterised a typical response to CSD, in which there is a brief drop in fluorescence followed by prolonged overshoot [126,127,128]. This relates to biphasic neurovasculature changes in mice, including an initial increased cerebral blood flow followed by a prolonged period of vasoconstriction [129,130,131]. This can account for fluorescence patterns in which there is an initial surge in NADH oxidation and ATP production, corresponding with ^31^P-MRS studies that exhibit increased V/Vmax [36,46]. This is followed by a prolonged period during which NADH is not utilised and has been hypothesised to result from hypoxia that occurs, when oxidative demand exceeds the oxygen supply [127]. The amplitude of change in NADH fluorescence during this pattern was one order of magnitude higher than the typical 2–4% changes resulting from physiological activity [127]. Suggesting that these oxidative metabolic transitions are pathophysiological during CSD. Additionally, repetitive CSD induction led to gradual decrease in fluorescence, indicating tissue compromise and suggesting that chronic innervation may lead to long-term damage [128].

These studies have suggested that hypoxia could be a substrate for CSD in brain tissue. Specifically in mouse, hypoxia is capable of lowering the threshold [127] and increasing the duration of CSD [132]. Interestingly in FHM mice, tissue oxygenation as a result of CSD reached anoxia levels (1.16 ± 0.78 mmHg), in comparison to wildtype mice which reached hypoxic levels (13.69 ± 3.45 mmHg) [133]. The authors suggested FHM mice used more energy to restore ionic gradients, hinting that those with hereditary migraine are more susceptible to metabolic perturbations in response to CSD. NADH measurements support findings that CSD is a metabolically challenging event and suggest that individuals with pre-existing mitochondrial deficits may have a lowered threshold for headache generating mechanisms.

#### 3.1.2. Glycolysis and Glucose Metabolism

Considering the significant evidence supporting the role of glucose metabolism and headache triggers, altering the glycaemic state in vivo can provide insights to the pathology underlying this susceptibility. Hyperglycaemia and hypoglycaemia were induced in rats by dextrose or insulin infusion [134]. Hyperglycaemia increased the threshold and reduced frequency of CSD events, whereas hypoglycaemia prolonged CSD duration and reduced KCl-induced CSD threshold by over 50% [134,135]. Another hypoglycaemic rat model induced by food restriction and insulin demonstrated faster CSD velocities than controls, which was reversed by injecting glucose [136]. Finally, CSD occurred spontaneously in insulin-treated rats (at blood glucose levels of 22 and 28 mg/dL) [137]. Apparent resistance to CSD in hyperglycaemia could be due to increased glucose availability which aids glycolysis and ATP production to maintain stimulus-induced rises in extracellular K^+^ [134]. The opposite therefore can be postulated for hypoglycaemia, which mirrors the glucose hypometabolism demonstrated in patients [68,69,138].

18FDG-PET tracing has also been utilised in rats in combination with microdialysis. This is able to provide an indication of extra-cellular availability of metabolites at the cortical surface during CSD. Unlike human studies, which exhibit hypometabolism in ictal periods, extracellular concentrations of glucose promptly decrease following CSD induction in rats, as it is rapidly utilized as an energy substrate (Figure 1) [139]. Cortical tissue exhibits a prolonged decrease in glucose content following CSD compared to contralateral unstimulated cortex, in conjunction with a rapid increase in lactate. Contradicting clinical studies, these changes in metabolites were accompanied by reduction in pH, indicating anaerobic glycolysis in response to CSD [140]. Moreover, 18FDG-PET confirmed an almost three-fold increase in lactate which was accompanied by extracellular acidification [141]. These studies further suggest an imbalance between ATP demand and oxygen availability as the tissue resorts to anaerobic processes. Utilisation of these alternative metabolic pathways may support ATP availability, and thus explain why no differences in ATP concentration were exhibited in ^31^P-MRS studies. Lactate was found to have a neuroprotective effect, reducing lesion size following excitotoxicity in ischemic stroke models [142]. Although it is a less energy efficient substrate, increased lactate in brain tissue may be a neuroprotective response to CSD.

### 3.2. Mitochondrial Studies in Animals

Whilst animal models allow the direct interrogation of mitochondrial integrity and function, few studies have been published utilising these methods. For instance, consumption of oxygen and extracellular acidification rates can be measured in vivo using the Seahorse XF analyser. This allows the quantification of mitochondrial and cytoplasmic respiratory pathways and has demonstrated alterations in a rat model of chronic migraine [143]. Using dural infusion of an inflammatory soup composed of histamine, serotonin, bradykinin and prostaglandin to induce trigeminal hypersensitivity, the model demonstrated a reduced spare respiratory capacity in trigeminal nucleus caudalis, a major component of the pain processing pathway [143]. Similarly, Clark-style oxygen electrodes able to measure oxygen consumption in mitochondria exhibited a significantly reduced mitochondrial membrane potential (the driving force for ATP synthesis) following CSD [144]. Although these findings mirror ^31^P-MRS studies indicating reduced mitochondrial energy reserves in migraine patients [17,45], it does not explain increased V/Vmax in these patients [36,46].

Furthermore, biochemical investigations have exhibited altered mitochondrial dynamics and biogenesis in trigeminal ganglion neurons following repeated inflammatory soup sensitization in rats [145]. Mitochondria exhibited fragmented structure, reduced DNA copy number, and alterations in mRNA and protein regulatory factors [145]. This disturbance was hypothesised to increase oxidative stress in tissue and potentially decrease the threshold for CSD events. Overall, these studies begin to pinpoint functional deficits in mitochondrial function, which may prove useful as therapeutic targets.

### 3.3. Supplementation of Animals

The use of Mg^2+^ supplementation in vivo has begun to unveil its role in headache pathology. Mg^2+^ has a vital function in NMDA receptors, imparting voltage sensitivity by blocking the receptor’s channel at resting membrane potentials and being removed during depolarization to allow Ca^2+^ influx and the activation of downstream signalling cascades. Trigeminal nerves are activated following direct stimulation of NMDA-receptors, making them important targets in migraine pain [146]. In mouse central neuron populations, the absence of Mg^2+^ increases the permeability of the receptor to cations [147], which may provide a mechanism for spontaneous CSD activity in rat hippocampal slices in low extracellular Mg^2+^ [49]. Mg^2+^ deficiency in headache patients, therefore, may be indicative of central sensitivity and the loss of NMDA-receptor blockade.

Unsurprisingly, these receptors have become an important target for migraine therapeutic development. MK-801 an NMDA-receptor antagonist, has demonstrated the ability to attenuate trigeminal nerve signalling in rats and cats [146,148]. Moreover, it has also demonstrated a role in abolishing CSD when induced in a rat brain [149]. Interestingly MgSO_4_ treatment was able to significantly reduce the number of CSDs induced when compared to untreated tissue [149]. MgSO_4_ treatment also increased latency times to anoxic depolarisation following cardiac arrest [149]. In rat brain slices, Mg^2+^ was able to improve recovery and maintain ATP levels during CSD in anoxic conditions, supporting its role in aiding respiration [150]. The ability of Mg^2+^ to block synaptic transmission and thereby reduce postsynaptic neuronal activation, lessens the metabolic burden required for homeostasis [150]. Moreover, the ability to perturbate spreading depression in anoxic conditions may be due to the role of Mg^2+^ in preserving energy mechanisms and ATP levels [149].

## 4. Conclusions

There is a wealth of clinical data characterising mitochondrial and metabolic dysfunction in migraine patients, similar to that of mitochondrial cytopathies [48]. Further in vivo investigation of these deficits demonstrates altered threshold for headache generating mechanisms including CSD. However, reproducibly pinpointing the exact deficits has been difficult to achieve both clinically and in vivo, which may contribute toward the mixed successes of nutraceutical trials. Understanding the metabolic deficiencies which increase susceptibility to CSD events may offer an approach to therapeutically recover mitochondrial energetic depletion, protect those susceptible to permanent tissue damage, and improve clinical phenotype.

## Data Availability

Not applicable.
